# The 7-valent pneumococcal conjugate vaccine elicits cross-functional opsonophagocytic killing responses to *Streptococcus pneumoniae* serotype 6D in children

**DOI:** 10.1186/1471-2334-13-474

**Published:** 2013-10-10

**Authors:** Hyunju Lee, Jung Hwa Cha, Moon H Nahm, Robert L Burton, Kyung-Hyo Kim

**Affiliations:** 1 Department of Pediatrics, Ewha Womans University School of Medicine, Seoul, Korea; 2 Center for Vaccine Evaluation and Study, Ewha Medical Research Institute, Ewha Womans University, Seoul, Korea; 3 Departments of Pathology and Microbiology, University of Alabama at Birmingham, Birmingham, Alabama 35294, USA

**Keywords:** *Streptococcus pneumoniae*, Heptavalent pneumococcal conjugate vaccine, Pneumococcal polysaccharide type 6

## Abstract

**Background:**

We investigated the immune response to serogroup 6 with the opsonophagocytic killing assay (OPKA) in children aged 12–23 months of age after immunization with the 7-valent pneumococcal conjugate vaccine (PCV7) containing serotype 6B.

**Methods:**

Blood samples were obtained from 59 children who had blood sampling for medical examination. Immunization status against PCV7 was confirmed by immunization records and samples were categorized according to immunization status into a booster, primary, or control group. The OPKA was performed for serotypes 6A, 6B, 6C, and 6D.

**Results:**

Subjects with no previous PCV7 immunization history showed opsonic activity for serogroup 6 in 5-30% (according to serotype). In subjects vaccinated with a 3-dose primary series, 81% showed opsonic activity for serotypes 6B and 6D, and 29% showed opsonic activity for serotypes 6A and 6C. Among subjects vaccinated with a booster dose, all subjects had opsonic activity against serotype 6B. Subjects in the booster group with opsonic activity against serotypes 6A, 6C, and 6D were 100%, 78%, and 89%, respectively.

**Conclusions:**

In subjects aged 12–23 months, an immune response is elicited after a primary series of immunizations with PCV7 for serotypes 6B and 6D, and a booster dose enhances a cross reactive immune response against serotypes 6A, 6C and 6D.

## Background

*Streptococcus pneumoniae* is a major cause of meningitis, pneumonia, and bacteremia in children and adults throughout the world. It is also a major cause of acute otitis media and sinusitis. With the introduction of the 7-valent pneumococcal conjugate vaccine (PCV7, Prevenar®, Pfizer Inc, Philadelphia, PA), a substantial decrease in invasive pneumococcal disease (IPD) due to serotypes included in the vaccine was seen among children and adults [1] since protection against *S. pneumoniae* is a serotype-specific immune response [2].

Up to several years ago, only serotypes 6A and 6B were grouped into serogroup 6 due to similarity in both chemical structure and serological properties of their capsular polysaccharides. Following the recent discovery of two new members, serogroup 6 now consists of 4 serotypes: 6A, 6B, 6C, and 6D [3,4]. Although *S. pneumoniae* serotype 6C is quite prevalent throughout the world, serotype 6D isolates are relatively rare in many countries [5-14]. For instance, the US CDC identified only two 6D isolates in its extensive multi-year surveys [15]. This rare finding of 6D in the US may be due to adequate cross-protection against 6D by PCV7, which contains serotype 6B PS that is similar to 6D PS in structure.

In contrast to the US, several epidemiologic studies in Korea reported high prevalence of serotype 6D, which accounts for up to 5-10% of serogroup 6 isolates [16,17]. They are found both among adults and children. This unusually high prevalence of 6D is unexpected since PCV7 is also widely used in Korea. Therefore it is possible that PCV7 does not elicit cross-protective antibodies against serotype 6D among Korean children. To investigate this possibility, we examined the immune response to serotypes 6A, 6C, and 6D, as well as 6B, with the opsonophagocytic killing assay (OPKA) in children aged 12–23 months of age after vaccination with PCV7.

## Methods

### Subjects

Subjects included in this study were children aged 12–23 months who had blood sampling for medical examination. Blood samples were obtained after informed consent. Immunization status against PCV7 was confirmed by immunization records and samples were categorized according to immunization status. Subjects with 3 primary doses and 1 booster dose of PCV7 were assigned to the booster group, subjects with 3 primary doses before 12 months of age were assigned to the primary group, and subjects with no vaccination history of PCV7 were assigned to the control group. Samples from 45 subjects who visited Kangnam CHA Medical Center from September to December 2006 have been described previously [18]. Serum samples from 14 subjects were additionally collected at Ewha Womans University Mokdong Hospital from November 2007 to February 2008. Children with underlying immunodeficiency disorders or a history of blood transfusion, immunoglobulin, or systemic steroid medication were excluded from the study. The study protocol was approved by the Institutional Review Board at Kangnam CHA Medical Center and Ewha Womans University Mokdong Hospital and was conducted in accordance with the Declaration of Helsinki and Good Clinical Practice guidelines. Informed written consent was obtained from all parents or legal guardians following a detailed explanation of the study.

### Opsonophagocytic killing assay

The opsonic indices (OI) of the samples were determined using the OPKA as previously described [19-23]. Target strains TREP6A, SPEC6B, SPEC6C, and SPEC6D (expressing capsule types 6A, 6B, 6C, and 6D, respectively) were derived from wild-type strains EF6796, BG25-9, BGO-2197, and MNZ920, respectively, and have been described previously [19,24]. Opsonic indices were defined as the serum dilution that kills 50% of bacteria and were determined by linear interpolation. In this study, all sera were diluted 5-fold before the assay due to limited amounts of sera. Because all sera were diluted 5-fold during the assay, the limit of detection was 20. A detailed protocol is posted on a website (http://www.vaccine.uab.edu).

### Statistical analysis

The analyses of serum antibody OI were based on logarithms of the OIs of all subjects. Geometric mean opsonic indices (GMI) were evaluated and two-sided 95% confidence intervals were determined for each pneumococcal serotype. Serum samples with OIs<20 were assigned a value of 10 for analysis purposes. The proportions of subjects achieving anti-pneumococcal OI ≥20 were determined. Reverse cumulative distribution curves were used to display percentages of children that achieved different OI to each of the 4 pneumococcal serotypes, 6A, 6B, 6C, and 6D.

## Results

### Characteristics of the subjects

A total of 59 subjects were included in the study. There were 18 subjects, 21 subjects, and 20 subjects in the booster, primary, and control group, respectively. The mean age of subjects in the booster group was 18.8 months (range 16–23 months) with a mean interval between last vaccination and sampling of 3.3 months (range 1–7 months). The primary group consisted of subjects with a mean age of 13.9 months (range 12–16 months) and the mean interval between the last vaccination and sampling was 7.2 months (range 4–9 months). The subjects in the control group were of a mean age of 14.5 months (range 12–16 months).

### Immune response to serogroup 6

**Figure 1:**
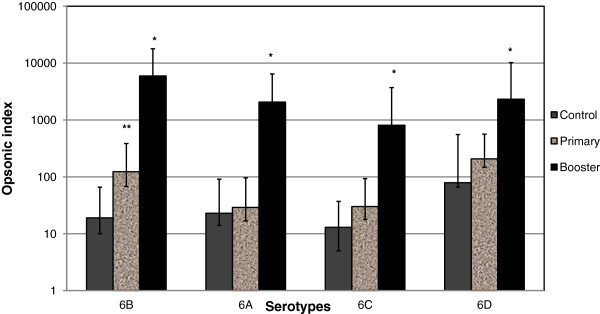
**Geometric mean opsonic indices of serogroup 6 in response to PCV7 according to vaccination status.** *P<0.05, Booster compared with primary and control groups; **P<0.05, Primary compared with control group PCV7; 7-valent pneumococcal conjugate vaccine.

The GMI for each serotype in serogroup 6 for each group of subjects is shown in Table 1. GMI of the booster group was higher compared with the primary and control groups for all 4 serotypes tested (Figure 1). When comparing the primary and control groups, GMI was higher for serotype 6B (*p*<0.05) in the primary group, however there was no difference between these groups for serotype 6A, 6C, and 6D.

**Table 1:** Geometric mean indices of serogroup 6 in response to PCV7 vaccine according to vaccination status

		**Booster (N=18)**	**Primary (N=21)**	**Control (N=20)**
6B	GMI	5,948	123^*,†^	19^*^
	95% CI	(2,969–11,917)	(55–263)	(9–47)
6A	GMI	2,065	29^*^	23^*^
	95% CI	(971–4,395)	(12–67)	(9–68)
6C	GMI	808	30^*^	13
	95% CI	(224–2,913)	(12–63)	(8–24)
6D	GMI	2,323	207^*^	79^*^
	95% CI	(683–7,988)	(57–357)	(21–476)

^*^P<0.05 compared with booster, ^†^*P*<0.05 compared with control.

PCV7; 7-valent pneumococcal conjugate vaccine, N; number, GMI; geometric mean index, CI; confidence interval.

**Figure 2:**
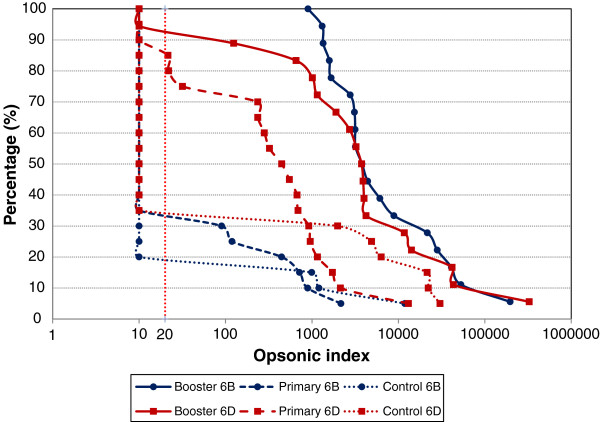
Reverse cumulative distribution curves of opsonic indices for serotypes 6B and 6D in booster, primary and control groups.

In the control group, 5-30% of the subjects had an OI > = 20, depending on serotype (Figure 2 and data not shown). In subjects who had previously been vaccinated with PCV7 as a primary series, and have not yet received the booster dose, 81% of the subjects had an OI ≥ 20 for serotypes 6B and 6D. However in the same subjects, only 29% had an OI ≥ 20 for serotypes 6A and 6C. After a booster dose, all subjects had OIs ≥ 20 for serotype 6B and 6A, while 89% and 78% had OIs ≥ 20 for serotypes 6D 6C, respectively.

## Discussion

Our study shows that PCV7 can elicit high levels of antibodies cross-reacting with serotypes 6A, 6C, and 6D in children 12–23 months of age. For serotype 6D, opsonic activity was demonstrable in 81% of the primary group and 89% of the booster group in these children. These high antibody responses are comparable with that of serotype 6B and actually higher than those for serotypes 6A and 6C. This finding is consistent with the paucity of 6D isolates found in the US, where PCV7 is used extensively. In contrast, 6D isolates are relatively more prevalent in Korea. Although there are no nationwide studies in Korea, according to a study of isolates collected from 1991–2008, 10.4% of serogroup 6 isolates were found to be serotype 6D [17]. A second study of isolates collected from 1997–2009 seemed to show an increase in 6D isolates in recent years [25]. However these studies were done on various populations of different ages. Therefore, we cannot directly determine the effect of PCV7 on serotype 6D. Further studies are needed to determine the effect of PCV7 on the epidemiology of 6D. If an increase in serotype 6D prevalence is real, it would be interesting to assess the OPA activity against 6D using isolates from Korea as target strains, to verify the high cross-protection immune response that was detected in this study.

In this study, 29% of the subjects in the primary group had a detectable OI for serotype 6C. This is consistent with the results of a previous study which found a seropositivity rate of 39% in infants following a 3-dose primary series (unpublished data). However, in the current study, 78% of subjects had a detectable OI for 6C in the booster group. Thus, the booster dose seems to significantly enhance cross-protection against 6C.

The seropositivity rate for serotype 6C in the primary group was relatively low compared with serotype 6D (29% vs 81%). This finding supports the epidemiologic phenomenon seen in 2004–2008 where an increase in invasive pneumococcal diseases due to serotype 6C was seen during a vaccine shortage [26]. Due to this vaccine shortage, many children were not able to receive a booster vaccination in 2004. With the absence of the booster dose, cross-protection was not sufficient for vaccine related serotypes [27,28].

The results of this study emphasize the importance of a booster dose for better protection of disease caused by both vaccine serotypes and vaccine-related serotypes. Compared to the control group which had no history of PCV7 vaccination, the immune responses to all serogroup 6 members were higher in primary group and highest in booster group. Infants are restricted in their ability to make diverse antibody variable regions and may produce antibodies with low avidity to cross-reactive serotypes [29]. However, it is known that multiple immunizations improve antibody maturation through somatic mutation resulting in high affinity antibody [30,31]. Our results of the cross-protective immune response according to vaccination dosages support this finding.

 There are limitations in this study. OPKA threshold for protection is identified as OI ≥ 8. Although there is controversy as to whether it is appropriate to apply a consistent value for all serotypes, Henckaerts et al. reported in a validation study of the OPKA, that OPKA sero-positivity (OI ≥ 8) correlated well with IPD effectiveness [32]. Since the threshold for OPKA was an OI ≥ 20 in this study, the results may be rather underestimated. Also, there were differences in age groups, where the mean age of children in the booster group was higher than the primary or control groups. Although none of the infants in the study had documented history of pneumococcal disease, this could have an effect on the antibody titers between groups, related to increase in natural exposure to pneumococcus with increase in age.

Compared to published data of the US, European, or Taiwanese infants, Korean infants had higher post-vaccination antibody titer to all serotypes in PCV7 [33]. Therefore further studies are needed in different population or ethnic groups. Moreover, this study is based on the immune response of PCV7. Recently conjugate vaccines containing more serotypes and different conjugate proteins have been introduced into the market [34,35]. The results of this study cannot be directly applied to these new vaccines, and further study is needed for different vaccine formulations. However PCV7 is still used in many countries. In this regard, the immune responses of PCV7 are of importance.

## Conclusion

We found that in subjects aged 12–23 months, an immune response is elicited after a primary series of immunizations with PCV7 for serotypes 6B and 6D and a booster dose enhances cross reactive antibody levels against serotypes 6A, 6C, and 6D.

## Abbreviations

OPKA: Opsonophagocytic killing assay; PCV7: 7-valent pneumococcal conjugate vaccine; IPD: Invasive pneumococcal disease; OI: Opsonic index; GMI: Geometric mean opsonic index; PS: Polysaccharide.

## Competing interests

This study was funded by RP-Grant 2011 of Ewha Womans University to HL and KHK and by the Korea Food and Drug Administration (11172KFDA360) to KHK. University of Alabama at Birmingham (UAB) owns intellectual property rights on the various reagents used for pneumococcal vaccine studies, and MHN and RLB are UAB employees.

## Authors’ contributions

Lee H managed the data and wrote the manuscript, Cha JW helped with statistical analysis, Burton RL carried out the immunoassays, Nahm MH participated in the preparation of the manuscript, and Kim KH participated in the design of the study and conceived of the study. All authors read and approved the final manuscript.

## Pre-publication history

The pre-publication history for this paper can be accessed here:

http://www.biomedcentral.com/1471-2334/13/474/prepub
